# Enhanced Antitumor Activity with Combining Effect of mTOR Inhibition and Microtubule Stabilization in Hepatocellular Carcinoma

**DOI:** 10.1155/2013/103830

**Published:** 2013-02-20

**Authors:** Qian Zhou, Chi Hang Wong, Cecilia Pik Yuk Lau, Connie Wun Chun Hui, Vivian Wai Yan Lui, Stephen Lam Chan, Winnie Yeo

**Affiliations:** ^1^Department of Clinical Oncology, The Chinese University of Hong Kong, Hong Kong; ^2^Cancer Drug Testing Unit, State Key Laboratory of Oncology in South China, Hong Kong Cancer Institute and Li Ka Shing Institute of Health Sciences, The Chinese University of Hong Kong, Hong Kong; ^3^Department of Otolaryngology, University of Pittsburgh School of Medicine, Pittsburgh, PA 15213, USA

## Abstract

Mammalian target of rapamycin (mTOR) and the microtubules are shown to be potential targets for treating hepatocellular carcinoma (HCC). PI3K/Akt/mTOR activation is associated with resistance to microtubule inhibitors. Here, we evaluated the antitumor activity by cotargeting of the mTOR (using allosteric mTOR inhibitor everolimus) and the microtubules (using novel microtubule-stabilizing agent patupilone) in HCC models. *In vitro* studies showed that either targeting mTOR signaling with everolimus or targeting microtubules with patupilone was able to suppress HCC cell growth in a dose-dependent manner. Cotargeting of the mTOR (by everolimus) and the microtubules (by patupilone, at low nM) resulted in enhanced growth inhibition in HCC cells (achieving maximal growth inhibition of 60–87%), demonstrating potent antitumor activity of this combination. *In vivo* studies showed that everolimus treatment alone for two weeks was able to inhibit the growth of Hep3B xenografts. Strikingly, the everolimus/patupilone combination induced a more significant antitumor activity. Mechanistic study demonstrated that this enhanced antitumor effect was accompanied by marked cell apoptosis induction and antiangiogenic activity, which were more significant than single-agent treatments. Our findings demonstrated that the everolimus/patupilone combination, which had potent antitumor activity, was a potential therapeutic strategy for HCC.

## 1. Introduction

Hepatocellular carcinoma (HCC) is the third most common cause of cancer-related deaths worldwide [1, 2]. Surgical resection and liver transplantation are the two mainstays of curative treatment for HCC, but can only be applied to the early stage of HCC [3, 4]. The majority of patients with HCC are not amenable to, or eventually failed, locoregional therapies and have to be considered for systemic treatment. Although sorafenib (a multikinase inhibitor of VEGFR, PDGFR, and Raf) has been approved for the treatment of HCC as the first-line therapy for unresectable HCC, the outlook of patients with advanced disease remains dismal [5, 6]. These reasons exemplify the need to design more effective therapeutic strategies. 

Everolimus (RAD001, Afinitor), a rapamycin analogue, is an oral mammalian target of rapamycin (mTOR) inhibitor. mTOR is a key effector in the PI3K/Akt/mTOR pathway and it plays a critical role in regulating cell proliferation, survival, and angiogenesis [7]. Everolimus has been approved for the treatment of papillary renal carcinoma, pancreatic neuroendocrine tumor, some types of breast cancer, and subependymal giant cell astrocytoma associated with tuberous sclerosis [8–11]. In HCC, a phase I/II study of everolimus has been conducted in patients with advanced HCC and antitumor activity was observed, with time to progression of 3.9 months and disease control rate of 44% [12]. 

However, to enhance the efficacy of everolimus [13, 14], evaluation for potential synergism with other classes of anticancer agents is warranted. Recent gene expression profiling studies suggested microtubules to be an important target for therapeutic intervention in HCC [15–17]. Furthermore, several studies demonstrated the involvement of mTOR pathway in resistance to microtubule-targeting chemotherapeutic agents [18, 19]. This led us to hypothesize that the cotargeting of mTOR and microtubules would be a potent therapeutic strategy for HCC. Indeed, in a previous study, we showed that combination of mTOR inhibitor temsirolimus and microtubule-targeting agent vinblastine had marked antitumor effect in HCC both *in vitro* and *in vivo* [20]. 

Patupilone, a macrocyclic polyketide, is a microtubule-stabilizing agent that belongs to the epothilone class. It binds to the *β*-tubulin subunit of microtubules [21, 22]. *In vitro* evidence indicates that patupilone is a more potent inducer of tubulin dimerization and is more effective in stabilizing preformed microtubules than taxanes [22, 23]. In HCC cell lines, patupilone is 4- to 130-fold more potent than taxanes [24]. Clinical studies of patupilone in solid tumor types including lung and ovarian cancers demonstrated high potency in its anticancer activity [25–27].

In the current study, we investigated the antitumor efficacy of everolimus in HCC, either alone or in combination with the novel microtubule-destabilizing agent, patupilone, in both *in vitro* and *in vivo* models of HCC. 

## 2. Materials and Methods

### 2.1. Reagents

Everolimus (RAD001/Afinitor) and patupilone (Epothilone B, EPO906) were obtained from Novartis Pharma (Basel, Switzerland) and dissolved in DMSO at a stock concentration of 10 mM and stored at −20°C. The following antibodies were used in the study: anti-mTOR, anti-pi-mTOR (ser2448), anti-Akt, anti-pi-Akt (ser473), anti-p70S6k, anti-pi-p70S6k (Thr389), anti-S6, anti-pi-S6 (ser240/244), anti-4E-BP1, anti-pi-4E-BP1 (ser65), anti-cleaved PARP (all from Cell Signaling Technology, Beverly, MA, USA), and anti-actin (Calbiochem, Nottingham, UK). 

### 2.2. Cell Culture

Human hepatocellular carcinoma cell lines Hep3B, HepG2, PLC/PRF/5, and SNU398 were obtained from the American Type Culture Collection (ATCC, Manassas, VA, USA) and Huh7 was obtained from Japanese Collection of Research Bioresources (JCRB, Japan). Hep3B, HepG2, Huh7, and PLC/PRF/5 were cultured in Dulbecco's modified Eagle medium with Glutamax-1 (HycClone, Logan, UT, USA) supplemented with 10% fetal bovine serum, FBS (HyClone). SNU398 was cultured in complete RPMI-1640 medium (HyClone) containing 10% FBS (HyClone). All cells were cultured under a humidified atmosphere of 5% CO_2_ at 37°C as previously described [17].

### 2.3. Cell Viability Assay

Cells (8000–18000 cells per well) were treated with either vehicle (DMSO) or increasing concentrations of everolimus (ranging from 0.1 nM to 20 *μ*M) or patupilone (ranging from 0.01 nM to 1 *μ*M) for 48 and 72 hrs. For combination treatment, cells were treated with increasing concentrations of everolimus and low concentration of patupilone (0.5 nM). Cell viability was determined by MTT assay as previously described [28]. The percentage growth inhibition was calculated as (OD_vehicle_ − OD_drug_)/OD_vehicle_ × 100%. The IC_50_ value was determined as the drug concentration at which half of the maximal growth inhibition was observed.

### 2.4. Western Blotting

Protein lysates were obtained as previously described [28]. Protein lysates (25–50 *μ*g) were separated by SDS-PAGE (sodium dodecyl sulfate-polyacrylamide gel electrophoresis) and transferred to nitrocellulose membranes. After primary and secondary antibody incubations, the signal was detected by autoradiography using SuperSignal West Pico Chemiluminescent Substrate (Thermo Fisher Scientific, Rockford, IL, USA).

### 2.5. HCC Xenograft Study

Four-to-six-week-old male athymic nude mice (nu/nu) were used for the establishment of HCC xenografts. All experiments were conducted under license from the Department of Health and according to animal ethics approval from the University Animal Experimentation Ethics Committee, the Chinese University of Hong Kong. HCC cells (3 × 10^6^ of Hep3B cells suspended in 200 *μ*L serum-free medium) were inoculated into the dorsal flanks of mice by subcutaneous injection. Mice were randomized into four groups. Treatments were started on day 20 after inoculation. The 4 treatment groups were (1) vehicle control, (2) everolimus alone (2.5 mg/kg, twice a week, orally), (3) patupilone alone (0.5 mg/kg, once a week, i.p.), and (4) a combination of everolimus and patupilone (two agents administered in an alternating schedule, not as mixed solution, with same doses and schedules as single agents alone). Tumor growth was monitored twice weekly and tumor volume was calculated using the formula of ((Length × Width^2^)/2) as previously published [29]. 

### 2.6. Immunohistochemistry and Microvessel Density (MVD) Determination

Immunohistochemistry was performed as previously described [30]. Tumor microvessels were stained with a rabbit anti-CD34 antibody (1 : 100 dilution, Santa Cruz Biotechnology). IHC score approach was applied to assess the intensity of staining for each xenograft specimen. The IHC score ranged from 1 to 4, 1 = −ve to weak, 2 = weak to moderate, 3 = moderate to strong, and 4 = strongest staining.

### 2.7. Statistical Analysis

All data were presented as mean ± SEM. Student's *t*-test (with Welch's correction) was performed using GraphPad Prism 4.0 software (GraphPad Software, Inc., San Diego, CA, USA). Results were considered as statistically significant if *P* < 0.05.

## 3. Results

### 3.1. Everolimus Inhibited HCC Cell Proliferation with Effective Inhibition of mTOR Signaling

**Figure 1:**
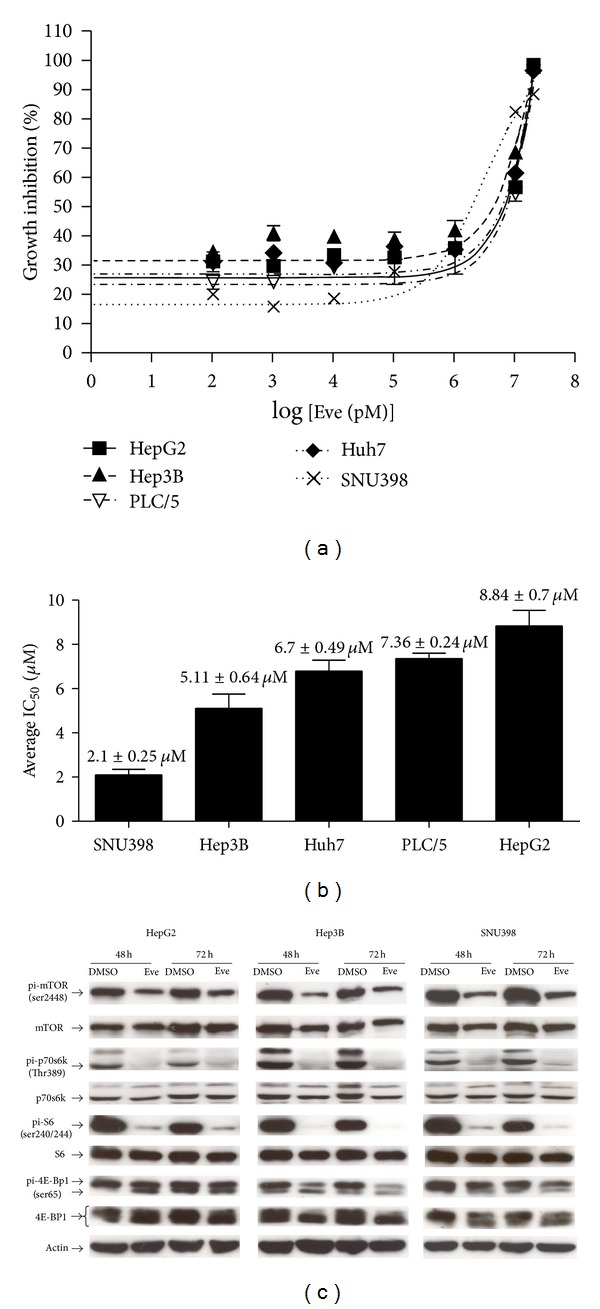
Everolimus inhibited proliferation and mTOR signaling in HCC cell lines. (a) Dose-dependent inhibition of HCC cell proliferation by everolimus. The effect of everolimus on cell viability was assessed by MTT assay. Dose-response curves of everolimus for all HCC cell lines were shown. Similar results were observed in 3 independent experiments. (b) Average IC_50_ values of everolimus in HCC cell lines. Cumulative results from 3 independent experiments were shown as mean ± SEM. (c) Everolimus inhibited the mTOR pathway in HCC cells. HepG2, Hep3B, and SNU398 cells (3 × 10^5^) were treated with 0.1 *μ*M everolimus (hereafter labeled as Eve) or DMSO control for 48 hrs and 72 hrs. The expression levels of the mTOR pathway components, pi-mTOR (ser2448), mTOR, pi-p70S6K (Thr389), p70S6K, pi-S6 (ser240/244), S6, pi-4E-BP1 (ser65), and 4E-BP1, and actin were examined by western blotting. Similar results were observed in 3 independent experiments.

To examine the effects of everolimus on HCC cell proliferation, five HCC cell lines (SNU398, Hep3B, HepG2, PLC/PRF/5, and Huh7) were treated with everolimus at increasing concentrations (0.1 nM–20 *μ*M). As early as 48 hrs upon treatment, everolimus was able to induce dose-dependent growth inhibition in all five cell lines tested, with a maximal achievable growth inhibition of ~90–95% at 20 *μ*M concentration. Among these HCC cell lines tested, SNU398 was the most everolimus-sensitive (average IC_50_ = 2.10 ± 0.25 *μ*M), while HepG2 was the most resistant one (average IC_50_ = 8.84 ± 0.70 *μ*M). The remaining three cell lines, Hep3B, Huh7, and PLC/5, had intermediate sensitivities (Figures 1(a) and 1(b)). 

Next, we examined the effects of everolimus on mTOR signaling in HCC cells. In HepG2, Hep3B, and SNU398 cells, everolimus (0.1 *μ*M) was able to elicit marked inhibition of mTOR signaling at 48 hrs, sustaining up to 72 hrs (Figure 1(c)). This was indicated by significant inhibition of phospho-mTOR (ser2448), as well as effective inhibition of its downstream effectors, including phospho-p70S6k (Thr389), phospho-S6 (ser240/244), and phospho-4E-BP1 (ser65) (Figure 1(c)). Our results showed that everolimus can abrogate mTOR activation and its downstream targets in HCC cells. It is noted that different extent of upregulation of phospho-Akt (ser473) was observed in the three cell lines upon everolimus treatment (Supplementary Figure 1(a) available online at http://dx.doi.org/10.1155/2013/103830), implicating a possible feedback upregulation of p-Akt by everolimus. 

### 3.2. Patupilone Inhibited HCC Cell Proliferation

**Figure 2:**
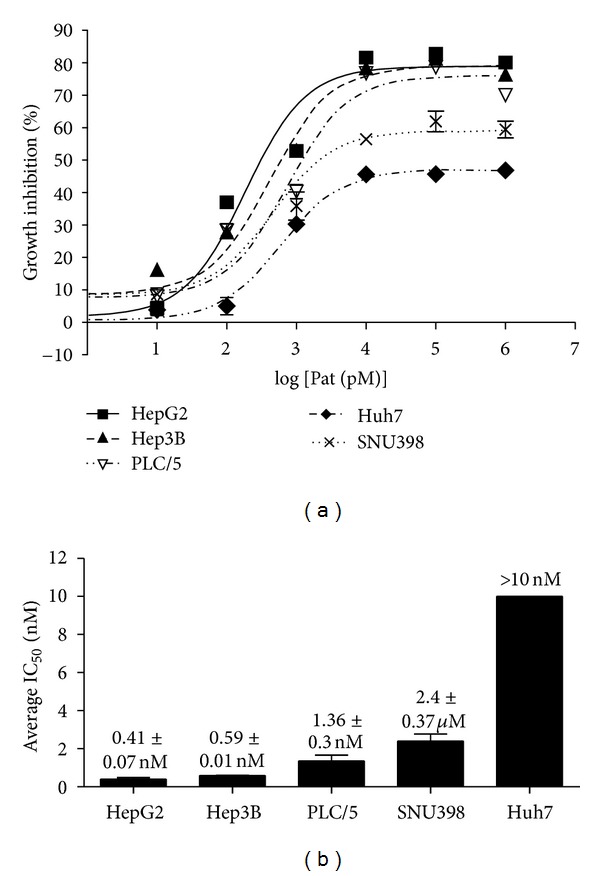
Patupilone inhibited proliferation in HCC cell lines. (a) Dose-dependent inhibition of HCC cell proliferation by patupilone. The effect of patupilone on cell viability was assessed by MTT assay. Dose-response curves of everolimus for all HCC cell lines were shown. Similar results were observed in 3 independent experiments. (b) Average IC_50_ values of patupilone in HCC cell lines. Cumulative results from 3 independent experiments were shown as mean ± SEM.

In present study, we examined the effects of patupilone on HCC cell proliferation in five HCC cell lines (SNU398, Hep3B, HepG2, PLC/PRF/5, and Huh7). Cells were treated with patupilone at increasing concentrations (0.01–1 *μ*M). Dose-dependent inhibition of cell proliferation was observed in all of these five cell lines after being treated with patupilone for 48 hrs. Among these HCC cell lines tested, HepG2 was the most everolimus-sensitive (average IC_50_ = 0.41 ± 0.07 *μ*M), while Huh7 was the most resistant one with IC_50_>10 *μ*M. The remaining three cell lines, Hep3B, SNU398, and PLC/5, had intermediate sensitivities (Figures 2(a) and 2(b)).

### 3.3. Enhanced Antitumor Activity of Everolimus/Patupilone Combination *In Vitro*

**Figure 3:**
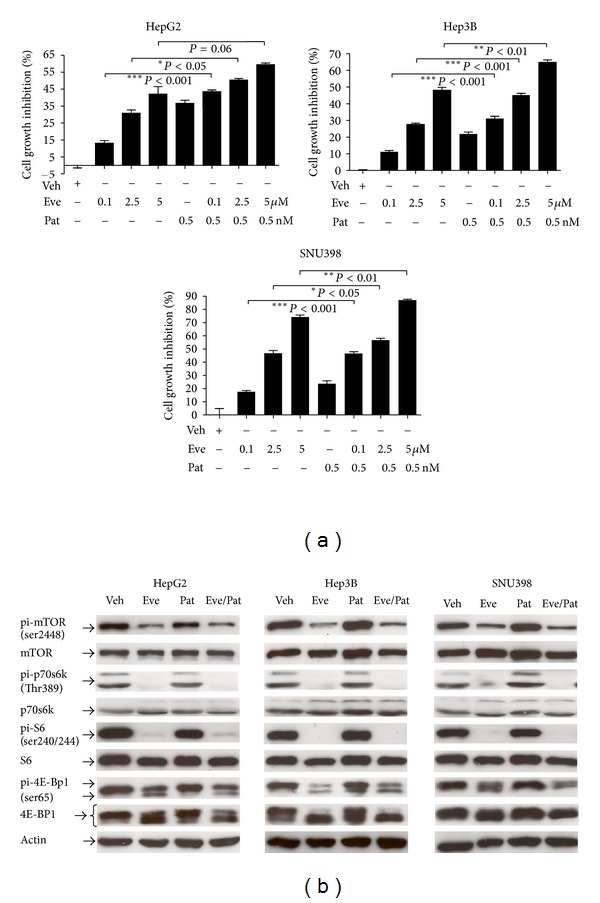
Enhanced antitumor activity of the everolimus/patupilone combination in HCC cell lines. (a) Effects of everolimus/patupilone in HCC cell lines. HepG2, Hep3B, and SNU398 cells (1 × 10^4^) were treated with various concentrations of everolimus in combination with 0.5 nM patupilone (Pat) for 24 hrs. Cell viability was assessed by MTT assay. Cumulative results from 3 independent experiments were shown as mean ± SEM (*P < 0.05, **P < 0.01, ***P < 0.001 versus everolimus-treated group). (b) The mTOR signaling in HCC cells was not further suppressed by the everolimus/patupilone combination treatment. HepG2, Hep3B, and SNU398 cells (3 × 10^5^) were treated with everolimus (0.1 *μ*M) and/or patupilone (Pat) (0.5 nM) for 24 hrs. The everolimus/patupilone combination is abbreviated as Eve/Pat hereafter. The expression levels of the mTOR pathway components, pi-mTOR (ser2448), mTOR, pi-p70S6K (Thr389), p70S6K, pi-S6 (ser240/244), S6, pi-4E-BP1 (ser65), and 4E-BP1, and actin were examined by western blotting. Similar results were observed in 3 independent experiments.

Studies in cervical and ovarian cancers revealed that activation of the PI3K/Akt/mTOR pathway is associated with resistance to microtubule-targeting agents, implicating a potential benefit of combined targeting of both the microtubules and the PI3K/Akt/mTOR pathway [18, 31]. Previous study by our group has shown synergistic antitumor effect of temsirolimus (an mTOR inhibitor) and vinblastine (a microtubule targeting agent) [20]. Here we examined the *in vitro* antitumor activity of everolimus/patupilone combination in HepG2, Hep3B, and SNU398 cells. As shown in Figure 3(a), the Hep3B cell line was only moderately sensitive to high dose of everolimus treatment at 48 hrs (with a maximal growth inhibition of only 48.09% even at 5 *μ*M concentration). Patupilone alone at low concentration (0.5 nM) only inhibited Hep3B proliferation by 20%. Strikingly, this low-dose patupilone with everolimus was able to enhance the growth inhibitory activity of everolimus (64.85 ± 1.46%, *n* = 3, *P* < 0.01) as early as 48 hrs. Similar findings were observed in the everolimus-sensitive SNU398 cells. A maximum growth inhibition of 86.93 ± 0.81% was observed in Huh7 cells with everolimus/patupilone combination (*n* = 3, *P* < 0.01) (Figure 3(a)). An enhanced growth inhibitory effect was also observed in the everolimus-resistant HepG2 cells, achieving 59.26 ± 1.07% maximal growth inhibition as early as 48 hrs (Figure 3(a)). Our findings in multiple HCC cell lines demonstrated marked therapeutic efficacy with such combination therapy. 

### 3.4. Everolimus/Patupilone Combination Elicited Potent Antitumor Activity *In Vivo*

**Figure 4:**
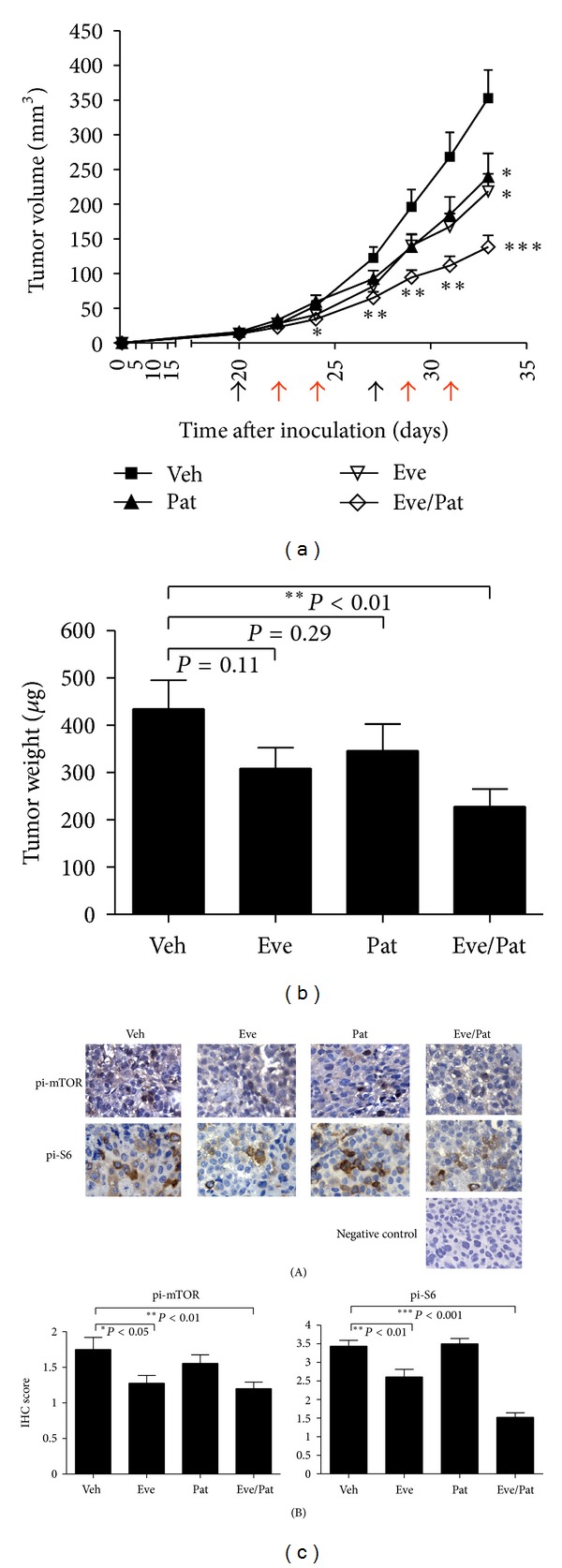
Potent antitumor effects of the everolimus/patupilone combination in* in vivo* models of HCC. Hep3B cells (3 × 10^6^ cells) were inoculated into nude mice by subcutaneous injection. Drug treatments were started on day 20 after inoculation. Mice received administration of drugs for two weeks (black arrow: patupilone i.p. injection; red arrow: everolimus orally given). (a) Treatment of mice with everolimus, patupilone, or combination suppressed tumor growth in established xenografts of Hep3B. Tumor growth was monitored twice weekly. Arrows indicated time of drug administration. The *in vivo* antitumor activity of everolimus/patupilone combination was more significant than either agent alone (*n* = 10 per group, *P < 0.05, **P < 0.01, ***P < 0.001 versus vehicle group). (b) Tumor weight of Hep3B xenografts in each group. The tumor weight of everolimus/patupilone combination group was significantly reduced (*n* = 10 per group, **P < 0.01 versus vehicle group). (c) The mTOR signaling in HCC cells was not further suppressed by the everolimus/patupilone combination treatment in Hep3B xenograft. Tumor xenografts were harvested, fixed, and stained for pi-mTOR and pi-S6 by immunohistochemistry. Representative images (400x magnification) were shown. Quantitation of pi-mTOR and pi-S6 staining using immunohistochemistry scoring was shown in (B) (*n* = 10 per group, *P < 0.05, **P < 0.01, ***P < 0.001 versus vehicle group).

The striking *in vitro* anticancer activity of this everolimus/patupilone combination compelled us to examine if this combination would be effective *in vivo*. Using established xenograft models of Hep3B (with intermediate everolimus-sensitivity, Figures 1(a) and 1(b)), we found that one week of everolimus treatment alone was able to inhibit the growth of Hep3B tumors, when compared to vehicle alone (average tumor volume of 81.75 ± 9.32 mm^3^ versus 122.66 ± 15.73 mm^3^ in vehicle alone; *n* = 15, *P* < 0.05) (Figure 4(a) and Table 1). An additional week of everolimus treatment also elicited significant change in tumor volume (average tumor volume of 218.56 ± 25.25 mm^3^ versus 352.97 ± 40.47 mm^3^ in the vehicle-treated group; *n* = 15, *P* < 0.05), consistent with the *in vitro* observation that these cells are moderately sensitive to everolimus (Figures 1(a) and 1(b)). Patupilone alone seemed to achieve a moderate degree of growth inhibition. However, as reported in an early study in which higher dose of patupilone was administered intraperitoneally [32], higher concentration of patupilone was lethal to mice in the present study (data not shown), thus limiting dose escalation of patupilone in mice. Consistent with the marked *in vitro* growth inhibitory activity of everolimus/patupilone combination, we found that this combination was able to inhibit Hep3B tumor growth significantly as early as 4 days after treatment (i.e., 24 days after tumor cell inoculation in Figure 4(a)). The most remarkable observation was that with only 2 weeks of treatment, the final tumor volume of the combination group was 138.57 ± 16.57 mm^3^ versus 352.97 ± 40.47 mm^3^ in the vehicle-treated group (~2.5-fold difference, *n* = 15, *P* < 0.001), 218.56 ± 25.25 mm^3^ in the everolimus only group, and 239.41 ± 33.81 mm^3^ in the patupilone only group (Figure 4(a) and Table 1). The final tumor weight of the combination group was 228.10 ± 37.20 *μ*g versus 434.70 ± 60.43 *μ*g in the vehicle-treated group (*n* = 15, *P* < 0.01), 308.60 ± 44.10 *μ*g in the everolimus-only group, and 346.10 ± 56.76 *μ*g in the patupilone-only group (Figure 4(b)). The treatment was tolerable by all groups with no deaths (data not shown).

### 3.5. Everolimus/Patupilone Combination Did Not Further Suppress mTOR Signaling in HCC Models

In order to examine the mechanism of such an enhanced antitumor activity of this combination, we examined the effects of this everolimus/patupilone combination on mTOR signaling pathway in HCC cells. As shown in Figure 3(b), everolimus/patupilone combination did not result in further suppression of mTOR signaling when compared to everolimus treatment alone, while patupilone alone did not alter mTOR signaling in HepG2, Hep3B, and SNU398 cells (Figure 3(b)). These results indicate that the enhanced antiproliferative effect of the everolimus/patupilone combination is probably unrelated to further suppression of mTOR signaling in HCC cells. Note that the feedback activation of Akt still persisted with the everolimus/patupilone combination treatment in all the three cell lines (Supplementary Figure 1(b)), suggesting that the efficacy of this combination was probably not due to inhibition of this Akt feedback in HCC cells. 

In fact, these *in vitro* findings were also confirmed in the respective *in vivo* models as well. As shown in Figure 4(c), pi-S6 and pi-mTOR levels were reduced in Hep3B tumors treated with either everolimus alone or with the combination, while patupilone did not suppress the two phosphoprotein levels.

### 3.6. Everolimus/Patupilone Combination-Induced Cell Apoptosis and Exerted Antiangiogenic Effect in HCC Models

**Figure 5:**
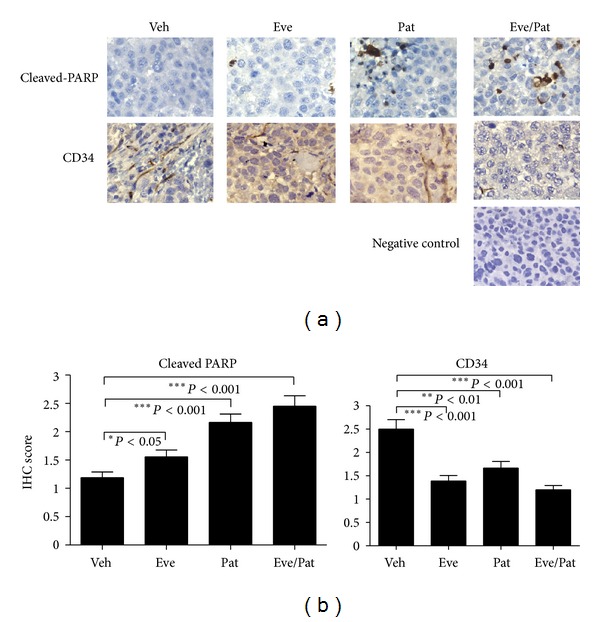
Everolimus/patupilone combination induced cell apoptosis and exerted antiangiogenic effect in HCC models. Tumor xenografts were harvested, fixed, and stained for cleaved PARP and CD34 by immunohistochemistry. Representative images (400x magnification) were shown. Quantitation of cleaved PARP and CD34 (microvessel density, MVD) staining was shown in (b) (*n* = 10 per group, *P < 0.05, **P < 0.01, ***P < 0.001 versus vehicle group).

Next, we examined if the marked antitumor activity of the combination was due to possible induction of apoptosis in these HCC models, as the PI3K/Akt/mTOR signaling pathway is known to be crucial for cell survival. As shown in Figure 5, PARP cleavage was readily detected (by IHC) in Hep3B tumors treated with everolimus and patupilone alone and further increased in tumors treated with the combination (Figures 5(a) and 5(b)). These results implied that the observed antitumor effect was at least partly mediated by cell apoptosis induced in the combination treatment.

In addition to the observed cell apoptosis induction in HCC xenografts, we also found that this combination was able to induce a significant reduction in microvessel density (MVD) in Hep3B models as compared to vehicle control (Figure 5), suggesting potent antiangiogenic activity of this combination in HCC models. As shown in Figure 5(b), administration of everolimus or patupilone alone in Hep3B xenografts for 15 days was able to inhibit MVD by 44.4% and 33.3%, respectively, while the combination inhibited MVD by 52% (*n* = 10, *P* < 0.001 versus vehicle control group). 

## 4. Discussion

In this study, we report the enhanced antitumor activity of cotargeting of mTOR (by everolimus) and the microtubules (by patupilone) in both *in vivo* and *in vitro* models of HCC, in which induction of cell apoptosis and inhibition of angiogenesis were detected. The observed additive to synergistic inhibitory effects of the everolimus/patupilone combination on HCC cell growth in multiple cell lines of HCC *in vitro *was further supported by the Hep3B xenograft model, where a potent antitumor and antiangiogenic effects were observed with only two cycles of this combination treatment. Our results indicate that the combination of everolimus with patupilone could be a highly effective regimen for HCC treatment, which warrants further clinical investigations in HCC patients.

We found that the HCC cell lines studied have demonstrated a similar sensitivity towards mTOR targeting by everolimus alone, with their IC_50_ ranging from 2.10 to 8.84 *μ*M. Previous studies in other cancers have indicated that mTOR targeting may elicit cytostatic effects rather than effective eradication of tumor cells [33, 34], suggesting that a combination of mTOR targeting with cytotoxic agents may be advantageous. Therefore, in search for a rational combination with everolimus, we decided to choose a combination with a microtubule-targeting agent, patupilone, based on the following evidence: (1) microtubule-targeting is believed to be a prominent druggable target in HCC [15–17], more importantly, (2) dual targeting of mTOR and microtubule by temsirolimus and vinblastine has recently shown sustained and potent antitumor effect in HCC models [20], and, lastly, (3) patupilone has been reported to be the most potent microtubule-targeting agent for HCC [35]. Indeed, we found that all the HCC cell lines that were tested were sensitive to patupilone, with the lowest IC_50_ being 0.41 nM. Further, when everolimus was combined with very low dose of patupilone (0.5 nM), enhanced effect was observed in HCC cell lines with a maximal achievable growth inhibition of about 70–90%. More interestingly, we found that the superior antitumor activity of the addition of patupilone in HCC models was not contributed to further suppression of mTOR signaling pathway compared with everolimus alone, implicating mTOR-independent effects on growth inhibition with this combination. 

When further investigating the mechanism involved, it was revealed that the combined treatment significantly induced cell apoptosis and suppressed angiogenesis, suggesting these two events to be the contributing mechanisms of the synergistic growth inhibition in HCC models. We found that PARP cleavage, which is a hallmark of cell apoptosis, was significantly increased in Hep3B xenograft tumors with the combined treatment versus vehicle control, although this effect seems to be mainly attributable to patupilone. This finding is consistent with the previous reports that mTOR targeting may only elicit cytostatic effects rather than cytotoxic effects [33, 34]. At the same time, microvessel density (MVD) was significantly reduced in tumors treated with the combination. In fact, the antiangiogenic effect by mTOR inhibitor and microtubule-targeting agent combination has been reported. Marimpietri et al. recently demonstrated that combination of rapamycin and vinblastine enhanced the therapeutic effect on human neuroblastoma growth, apoptosis, and angiogenesis [36]. Moreover, rapamycin/vinblastine combination was found to exert antiangiogenic effects in an endothelial cell line EA.hy926 [37]. A previous study by our group has also shown that temsirolimus/vinblastine combination had marked antiangiogenic effect in HCC. In the current study, we further demonstrated the antiangiogenic effect with mTOR/microtubule targeting.

Everolimus is currently undergoing a phase III clinical trial in HCC. The earlier phase I/II study of everolimus has shown modest antitumor activity, with median progression-free survival of 3.8 months and overall survival of 8.4 months in patients with advanced HCC [38]. As a novel microtubule-targeting agent, patupilone has only shown modest antitumor effect as a single agent in a phase II study conducted in advanced HCC, with progression-free survival of 3 months and disease stabilization rate of 44% [39]. Based on the data from the current study, we were able to show for the first time that combination of a very low dose of patupilone with everolimus was able to result in a much stronger antitumor effect when compared to either of the single agents alone in HCC models.

## 5. Conclusions

In conclusion, our study demonstrated that the combination of everolimus with low dose of patupilone could be a highly effective regimen for the treatment of HCC. Clinical investigation into the role of such combination in HCC patients is warranted.

## Supplementary Material

Supplementary Figure 1: (a) Everolimus treatment induced Akt phosphorylation in HCC cells. HepG2, Hep3B and SNU398 Cells (3×105) were treated with 0.1*μ*M everolimus or DMSO control for 48 hrs and 72 hrs. The expression levels of pi-Akt (Ser473), Akt and actin were assessed by Western blotting. Similar results were observed in 3 independent experiments. (b) Everolimus/patupilone combination did not suppress Akt phosphorylation in HCC cells. HepG2, Hep3B and SNU398 cells (3×105) were treated with everolimus (0.1 *μ*M) and/or patupilone (Pat) (0.5nM) for 24 hrs. The expression levels of pi-Akt (Ser473), Akt and actin were assessed by Western blotting. Similar results were observed in 3 independent experiments.

## Figures and Tables

**Table 1:** Average tumor volume and tumor weight during treatments in Hep3B xenograft model.

Treatments	Average tumor volume (mm^3^)		Average tumor weight (*μ*g)
	Week 1	Week 2	End of experiment
Veh	122.66 ± 15.73	352.97 ± 40.47	434.70 ± 60.43
Eve	81.75 ± 9.32	218.56 ± 25.25	308.60 ± 44.10
	(*P < 0.05)	(*P < 0.05)	(*P* = 0.11)
Pat	92.40 ± 12.03	239.41 ± 33.81	346.10 ± 56.76
	(*P* = 0.14)	(*P < 0.05)	(*P* = 0.29)
Eve/Pat	65.14 ± 8.62	138.57 ± 16.57	228.10 ± 37.20
	(**P < 0.01)	(***P < 0.001)	(**P < 0.01)

*P < 0.05 versus vehicle control group; **P < 0.01 versus vehicle control group; ***P < 0.001 versus vehicle control group.

## Abbreviations

HCC: Hepatocellular carcinoma
mTOR: Mammalian target of rapamycin
4E-BP1: eIF4E binding protein
MVD: Microvessel density
MDR: Multidrug resistant gene.
