# Supplementary material for: Enhanced Antitumor Activity with Combining Effect of mTOR Inhibition and Microtubule Stabilization in Hepatocellular Carcinoma
Source: Int J Hepatol. 2013 Feb 20;2013:103830. doi: 10.1155/2013/103830 (PMC3590758; doi:10.1155/2013/103830)

**Supplementary Figure 1. (a) Everolimus treatment induced Akt phosphorylation in HCC cells.** HepG2, Hep3B and SNU398 Cells (3×10^5^) were treated with 0.1μM everolimus or DMSO control for 48 hrs and 72 hrs. The expression levels of pi-Akt (Ser473), Akt and actin were assessed by Western blotting. Similar results were observed in 3 independent experiments. **(b) Everolimus/patupilone combination did not suppress Akt phosphorylation in HCC cells.** HepG2, Hep3B and SNU398 cells (3×10^5^) were treated with everolimus (0.1μM) and/or patupilone (Pat) (0.5nM) for 24 hrs. The expression levels of pi-Akt (Ser473), Akt and actin were assessed by Western blotting. Similar results were observed in 3 independent experiments.


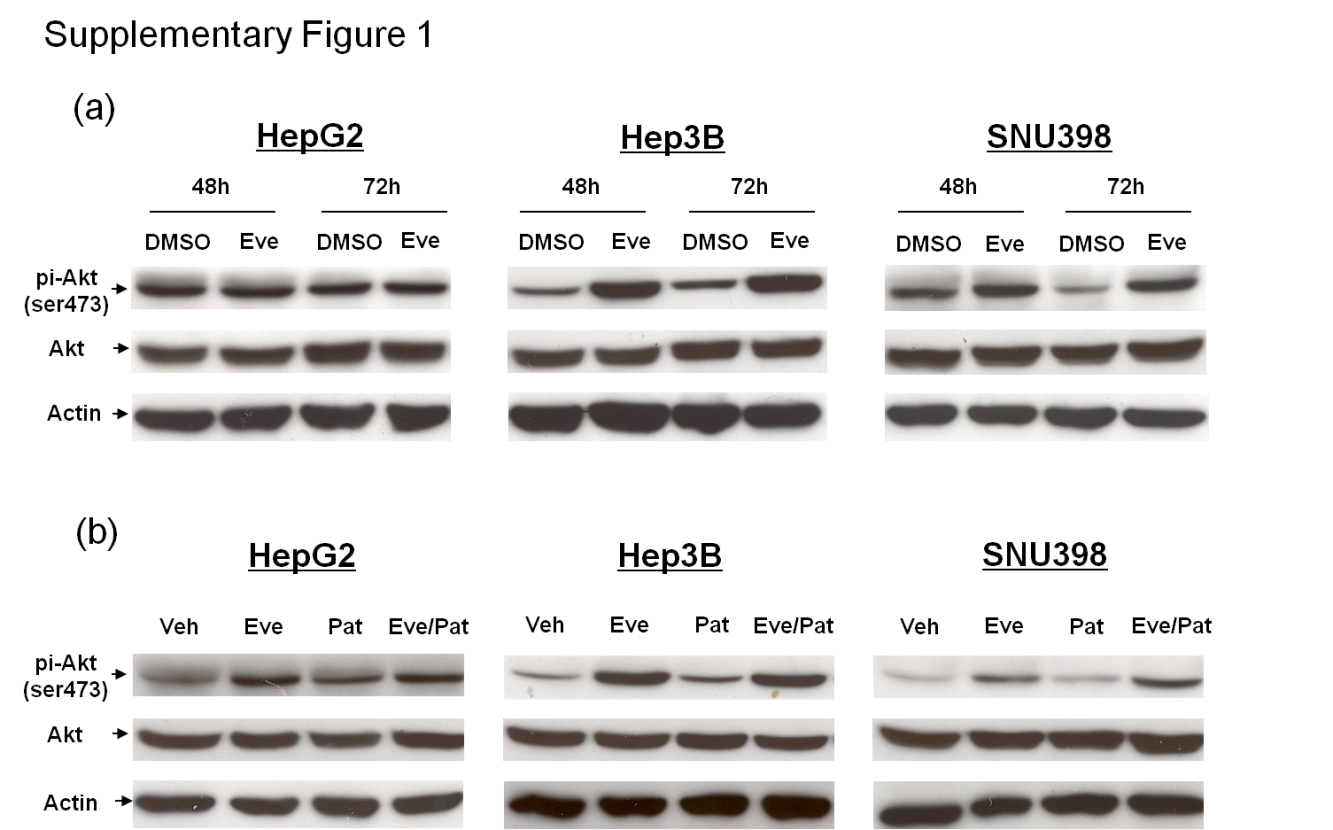

Supplement: Supplementary file 1 — Supplementary Figure 1: (a) Everolimus treatment induced Akt phosphorylation in HCC cells. HepG2, Hep3B and SNU398 Cells (3×10^5^) were treated with 0.1μM everolimus or DMSO control for 48 hrs and 72 hrs. The expression levels of pi-Akt (Ser473), Akt and actin were assessed by Western blotting. Similar results were observed in 3 independent experiments. (b) Everolimus/patupilone combination did not suppress Akt phosphorylation in HCC cells. HepG2, Hep3B and SNU398 cells (3×10^5^) were treated with everolimus (0.1 μM) and/or patupilone (Pat) (0.5nM) for 24 hrs. The expression levels of pi-Akt (Ser473), Akt and actin were assessed by Western blotting. Similar results were observed in 3 independent experiments. [file 103830.f1.docx]
